# Altered Lipolytic Protein Expression in Tumor-Associated Adipose Tissue Correlates with Aggressive Breast Cancer Features

**DOI:** 10.3390/cancers18183001

**Published:** 2026-09-16

**Authors:** Priscila Pagnotta, Tomás González-Garello, Cecilia Lotufo, María Luján Crosbie, Natalia Santiso, Anabela Ursino, Celeste Frascarolli, Alicia Amato, Rubén Dreszman, Camila Ramos Quintana, Juan Carlos Calvo, Judith Toneatto

**Affiliations:** 1 Instituto de Biología y Medicina Experimental (IBYME), Consejo Nacional de Investigaciones Científicas y Técnicas (CONICET), Vuelta de Obligado 2490, Buenos Aires 1428, Argentina; priscila.pagnotta@gmail.com (P.P.); cecilialotufo@yahoo.com.ar (C.L.); camquint26@gmail.com (C.R.Q.); juancalvo@ibyme.conicet.gov.ar (J.C.C.); 2 Departamento de Química Biológica, Facultad de Ciencias Exactas y Naturales, Universidad de Buenos Aires, Avenida Intendente Cantilo, Buenos Aires 1428, Argentina; 3 Instituto de Cálculo (IC), Facultad de Ciencias Exactas y Naturales, Universidad de Buenos Aires, Avenida Intendente Cantilo, Buenos Aires 1428, Argentina; tomas21.gg@gmail.com; 4 Complejo Médico Policial Churruca-Visca, Uspallata 3400, Buenos Aires 1437, Argentina; crosbieml@hotmail.com (M.L.C.); natalia_santiso@hotmail.com (N.S.); anabelaursino@yahoo.com.ar (A.U.); frascarolliceleste@gmail.com (C.F.); aliciaamato35@gmail.com (A.A.); 5 Clínica de Microcirugía, Bulnes 1813, Buenos Aires 1425, Argentina; rdreszman@fibertel.com

**Keywords:** hormone receptor-positive (HR+) breast cancer, tumor-associated adipose tissue, perilipin 1, ATGL, HSL, clinicopathological characteristics

## Abstract

Background: Adipose tissue (AT) has emerged as a metabolically active component of the breast tumor microenvironment that contributes to breast cancer (BC) progression. Adipocytes release adipokines and lipids that influence tumor growth; however, the specific lipolytic proteins involved in these interactions remain poorly characterized. Methods: In this study, we analyzed the expression of perilipin 1, adipose triglyceride lipase (ATGL), and hormone-sensitive lipase (HSL) by Western blot and immunofluorescence in adipose tissue explants from BC patients and healthy donors, evaluating their associations with clinicopathological features related to BC prognosis. Results: We found that perilipin 1 expression was increased in AT from patients with aggressive tumor characteristics, including advanced stage, premenopausal status, and obesity. In contrast, ATGL and HSL levels were reduced in specific patient subgroups, particularly in obese and postmenopausal patients and in tumor-adjacent AT from invasive lobular carcinoma. Conclusions: Together, these findings indicate that the expression of proteins involved in the regulation of lipolysis is influenced by both tumor proximity and clinicopathological characteristics, suggesting alterations in the molecular machinery governing lipid droplet regulation and lipid mobilization within tumor-associated AT. The observed associations identify perilipin 1, ATGL, and HSL as potential candidate markers of tumor-associated AT alterations and provide further insight into tumor–adipocyte interactions in BC. Despite the exploratory nature of this study, the observed associations provide a basis for further investigation into their potential biological and clinical relevance in BC.

## Simple Summary

Breast cancer grows within a complex environment that includes adipose tissue (AT), a major component of the breast. Increasing evidence indicates that adipocytes surrounding the tumor actively influence cancer behavior by releasing molecules and nutrients that support neoplastic growth. However, it remains unclear how proteins involved in fat breakdown are altered in tumor-associated AT and whether these changes are linked to disease progression. In this study, we examined key lipolytic proteins in breast AT from patients with breast cancer and from healthy women, and found their expression to be associated with aggressive tumor features and with certain patient characteristics, such as obesity and menopausal status. These findings suggest that altered AT metabolism may contribute to breast cancer progression and highlight lipolytic proteins as potential biomarkers and therapeutic targets within the tumor microenvironment.

## 1. Introduction

Cancer remains a major global health challenge despite substantial advances in diagnosis and treatment [1]. Among women, breast cancer has the highest incidence of all malignancies and represents the leading cause of cancer-related death worldwide. Its marked clinical and molecular heterogeneity is a major challenge for clinical management [2], motivating the search for new pathways into the complexity of this disease. In particular, the tumor microenvironment has emerged as a critical factor in cancer progression, acting as an active and dynamic component that influences tumor growth and therapeutic response [3].

The breast tumor microenvironment is largely composed of adipose tissue (AT), an endocrine organ that contributes to systemic metabolic homeostasis through secretion of bioactive molecules and remodeling of the extracellular matrix [4]. The cellular components of AT—adipocytes, stromal cells, endothelial cells, fibroblasts, and immune cells—collectively shape the metabolic landscape of this compartment [3,4]. The contribution of stromal cells to cancer progression has been extensively studied [5]. However, adipocytes have only recently been considered key regulators of tumor metabolism; these cells can promote cancer progression by secreting adipokines, cytokines, and lipids that modulate tumor cell metabolism and behavior [5,6].

Conversely, tumor cells release signaling molecules that can alter adipocyte phenotype and function, contributing to bidirectional tumor–adipocyte communication. This intercellular crosstalk is mediated by multiple mechanisms, including soluble factors, lipids, and extracellular vesicles, which are considered important mediators of communication between tumor and stromal cells in breast cancer [7].

Regulation of lipid metabolism is one of the processes potentially affected by tumor–adipocyte crosstalk. Among the multiple metabolic pathways, lipolysis is particularly relevant due to its direct contribution to the tumor lipid supply. Lipolysis involves the sequential hydrolysis of triglycerides stored in lipid droplets, generating free fatty acids and glycerol that may serve as metabolic substrates for tumor cells [6,8].

This process is tightly regulated by proteins including perilipin 1, adipose triglyceride lipase (ATGL), and hormone-sensitive lipase (HSL), which play central roles in lipid droplet regulation and triglyceride hydrolysis. Under basal conditions, ATGL, in association with CGI-58, catalyzes the hydrolysis of triglycerides into diacylglycerol [8,9], which is subsequently hydrolyzed by HSL [10]. Perilipin 1, a major regulator of lipid droplet dynamics, restricts the access of lipases to stored triglycerides under basal conditions, whereas its phosphorylation by protein kinase A (PKA) promotes lipid droplet remodeling and facilitates lipolysis [11]. Alterations in the regulation of lipolysis have been implicated in metabolic disorders such as obesity and insulin resistance, and accumulating evidence suggests that dysregulation of lipid mobilization may also influence tumor progression [8,12,13].

Our previous studies have established a bidirectional relationship between breast cancer cells and AT within the tumor microenvironment (tumor-associated adipose tissue, TAAT). We demonstrated that soluble factors released by TAAT induce changes in adipocyte phenotype, including increased lipolysis, morphological remodeling, and acquisition of beige-like features [14]. Conversely, beige adipocytes and soluble factors released by AT adjacent to the tumor can modulate breast cancer cell behavior, promoting proliferation and migration and altering the expression of tumor-related markers [15,16,17]. We further identified that FABP4 and vimentin are altered in AT adjacent to the tumor, and we showed that this compartment releases soluble factors that differentially affect tumorigenic traits according to breast cancer subtype [17]. Together, these findings support a dynamic bidirectional crosstalk between AT and breast cancer cells and highlight the importance of AT in shaping the tumor microenvironment. However, the expression of key proteins involved in lipid droplet regulation and lipolysis in TAAT, and their relationship with tumoral and clinical characteristics, remain poorly defined. Based on this rationale, we hypothesized that AT within the breast tumor microenvironment exhibits altered expression patterns of key proteins involved in lipid droplet regulation and lipolysis, and that these alterations are associated with tumor proximity and with patient- and tumor-related characteristics relevant to breast cancer progression. To test this hypothesis, we analyzed human breast AT explants from a cohort of patients with hormone receptor-positive (HR+) breast cancer; these samples were classified according to their proximity to the tumor: adjacent explants (<2 cm from the tumor margin) and distant explants (>2 cm from the tumor margin). We evaluated the expression of perilipin 1, ATGL, and HSL and analyzed their associations with clinicopathological characteristics.

## 2. Materials and Methods

### 2.1. Reagents

Reagents were purchased from Sigma-Aldrich/Merck Chemical Co. (St. Louis, MO, USA); molecular biology reagents were obtained from Invitrogen (Carlsbad, CA, USA). Antibodies were purchased from Abcam (Cambridge, MA, USA) and Santa Cruz Biotechnology (Dallas, TX, USA).

### 2.2. Sample Collection and Handling

**Scheme 1:**
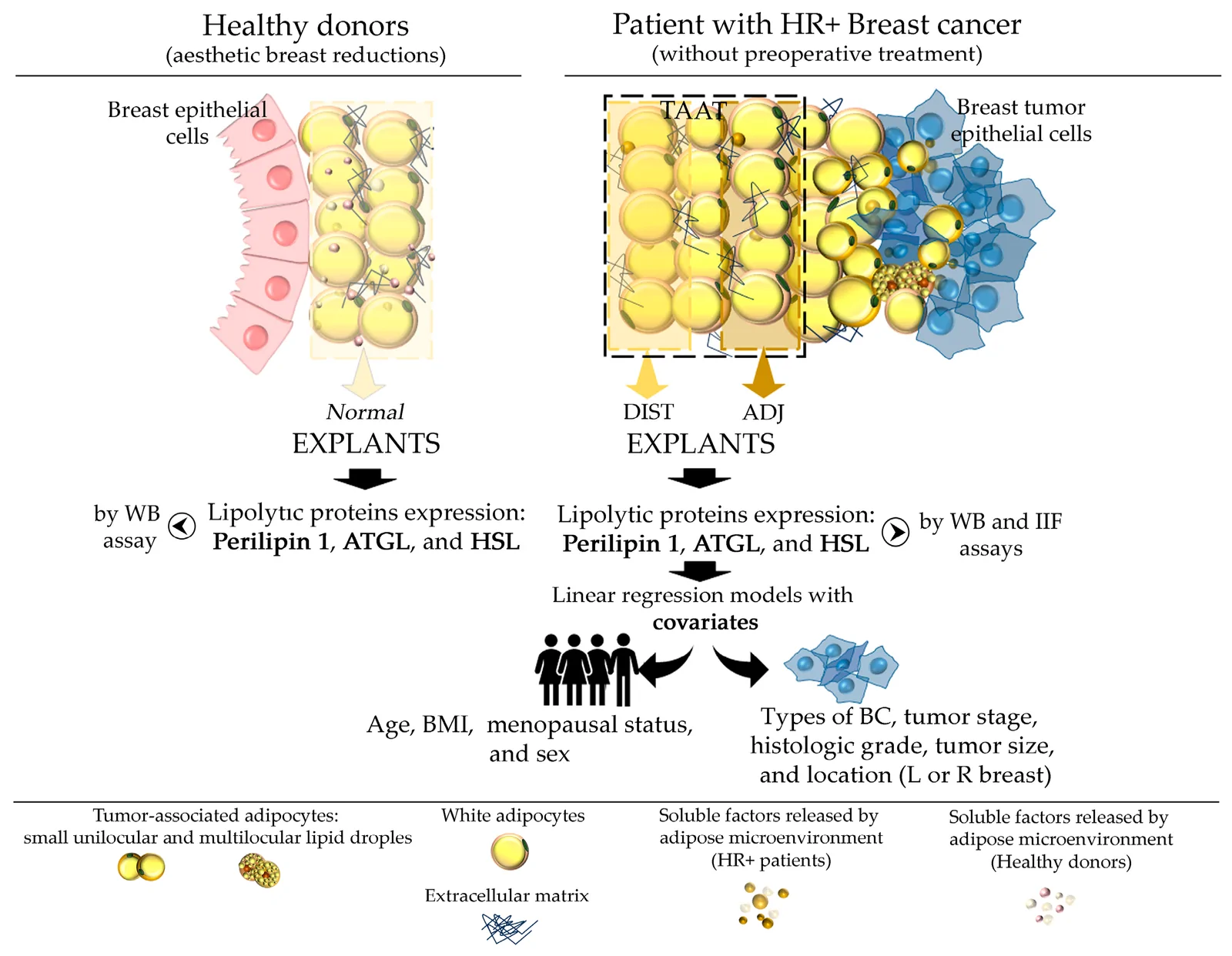
Schematic experimental approach. A diagram illustrating the methodology employed in this study, highlighting the analysis of protein expression levels for lipolytic proteins: perilipin 1 and lipases (ATGL and HSL) in different areas of the AT within the breast cancer microenvironment and in normal breast AT. The analysis was performed on AT explants from patients with hormone receptor-positive breast carcinoma who had not received preoperative treatment (adjacent to and distant from the tumor; **right** diagram) and from healthy female donors (**left** diagram) using WB and IIF. ADJ, *adjacent* breast explants (AT < 2 cm from the tumor); AT, adipose tissue; BC, breast cancer; BMI, body mass index; DIST, *distant* breast explants (AT > 2 cm from the tumor); HR+, hormone receptor-positive breast cancer (estrogen receptor-positive, progesterone receptor-positive and HER2-negative); IIF, indirect immunofluorescence; L, left breast; R, right breast; TAAT, tumor-associated adipose tissue (includes *adjacent* and *distant* explants); WB, Western blot.

The study was approved by the ethics committees of the Churruca-Visca Police Medical Center and the Institute of Biology and Experimental Medicine (IBYME) (CE025). Women undergoing mastectomy at the Churruca-Visca Police Medical Center provided informed consent under a standard tissue acquisition protocol. Human AT from breast explants of patients with hormone receptor-positive (HR+) breast carcinoma who had not received preoperative treatment (n = 43) was collected from sites adjacent to and distant from the tumor (defined as up to 2 cm away [termed *adjacent*] and over 2 cm away [termed *distant*], respectively). Breast explants from healthy women undergoing aesthetic surgery served as normal AT controls (termed *normal*, n = 11); informed consent was also obtained from these patients. The experimental approach is outlined in Scheme 1.

Samples were handled following a procedure previously described in the literature [18]. In brief, samples were transported to the laboratory in phosphate-buffered saline (PBS) containing 50 µg/mL gentamicin (Gentamicin sulfate, Richet Laboratory, Buenos Aires, Argentina) and processed in a laminar flow hood within 2 h after surgery. Paraffin blocks and snap-frozen samples were prepared for each case. Frozen samples were stored at −80 °C and subsequently processed for Western blot (WB), as described in Section 2.3 and Section 2.4. The tissue was fixed in 4% buffered formaldehyde and embedded in paraffin; the blocks obtained were sectioned into 3 μm thick slices for indirect immunofluorescence assay (IIF), as described in Section 2.5.

### 2.3. Total Protein Lysates from Tissue Explants

Protein lysates were prepared from frozen *normal*, *adjacent*, and *distant* explants. The samples were homogenized in RIPA buffer [50 mM Tris-HCl pH 7.5; 150 mM NaCl; 1% Igepal CA-630 (NP 40); 0.5% sodium deoxycholate; and 0.1% sodium dodecyl sulfate (SDS)] supplemented with protease and phosphatase inhibitors (1 mL/200 mg of tissue) using a Bio-Gen PRO200 homogenizer (PRO Scientific, Inc., Oxford, CT, USA) in ice-cooled tubes. Subsequently, the samples were centrifuged at 13,400× *g* for 15 min at 4 °C, the supernatant was collected, and a second centrifugation was performed for 5 min under the same conditions. Protein concentration was determined using the DC Protein Assay kit (Bio-Rad Laboratories, Hercules, CA, USA). The protein lysates were stored at −80 °C.

### 2.4. Western Blot Assay

Protein extracts (25–50 μg) were separated on 12% SDS-PAGE gels following the procedure previously described [16]. Briefly, proteins were separated by electrophoresis at 100 V until the tracking dye front reached the bottom of the gel and were subsequently transferred onto PVDF membranes. Membranes were blocked with 1% bovine serum albumin (BSA, Sigma-Aldrich; 10735086001) in Tris-buffered saline containing 0.05% Tween 20 (TBS-T) for 1 h and incubated overnight at 4 °C with the corresponding primary antibodies. After washing, the membranes were incubated with horseradish peroxidase-conjugated secondary antibodies for 90 min at room temperature. Protein detection was performed using a chemiluminescence reaction with an ECL solution containing luminol (Sigma-Aldrich; 123072), p-coumaric acid (Sigma-Aldrich; C9008), and H_2_O_2_. Band intensity was quantified using ImageJ software (version 1.52p; NIH, Bethesda, MD, USA). The specific protein bands quantified were: perilipin 1 (~62 kDa), the ATGL doublet (~55 kDa), and HSL (~90 kDa). Actin was used as a loading control. The antibodies and dilutions used in this assay were as follows: Actin (I19 sc-1616R, 1:1000) and HSL (sc-27954, 1:1500) from Santa Cruz Biotechnology (Santa Cruz, CA, USA); ATGL (ab240381, 1:500) and perilipin 1 (ab3526, 1:5000) from Abcam; and rabbit anti-IgG-HRP (A0545, 1:6000) from Sigma-Aldrich. Precision Plus Protein™ All Blue Standards (Bio-Rad Laboratories) were used to determine protein molecular weights. Membranes were sectioned horizontally according to the migration pattern of the molecular weight markers, enabling the separate immunodetection of high- and medium-molecular-weight targets with their respective primary antibodies. The numbers of adipose explants used in the WB assays were n *normal* = 11, n *adjacent* = 23, and n *distant* = 20. Characteristics of healthy donors and breast cancer patients are summarized in Tables S1 and S2 of the Supplementary Material, respectively. The original, unprocessed WB membranes are included in Figure S1 of the Supplementary Material.

### 2.5. Indirect Immunofluorescence (IIF)

Sections of AT were deparaffinized at 60 °C for 2 h, then treated with xylene (three 10-min washes), followed by 5-min washes in 100%, 96%, 90%, and 70% ethanol, and TBS. Antigen retrieval was then performed by boiling the samples in citrate buffer (pH 6). Following blocking (2% BSA in TBS, 20 min), the sections were incubated overnight at 4 °C in a humidified chamber with the corresponding primary antibodies, and subsequently incubated with the corresponding secondary antibody at room temperature for 45 min. The specimens were mounted with Vectashield (#H 1000, Vector Laboratories, Inc., Burlingame, CA, USA), and images were acquired using a Spinning Disk DSU IX83 microscope (Olympus Corporation, Tokyo, Japan) with a 10× objective. Images were analyzed using ImageJ with an automated script designed to identify intact adipocytes and quantify fluorescence intensity per cell based on size and circularity. Secondary antibody-only controls were included to rule out non-specific binding, and autofluorescence controls were performed to assess background fluorescence from the samples. The maximum number of representative fields per sample was analyzed to ensure reliable quantification, including intact adipocytes identified by the automated script. Only regions with homogeneous labeling were considered, and areas along the cut edge were excluded. For each explant, the average fluorescence intensity per adipocyte was calculated and used for statistical analyses. The antibodies and dilutions used in the assay were: Alexa Fluor 555 nm (ab150074, 1:400), ATGL (ab240381, 1:100), HSL (ab45422, 1:200), and perilipin 1 (ab3526, 1:500). The numbers of adipose explants used in the IIF assays were n *adjacent* = 19 and n *distant* = 17.

### 2.6. Statistical Analysis

Statistical analyses used to characterize the expression of each lipolytic protein were based on analysis of variance (ANOVA). Given the paired structure of the data, models involving two or more observations from the same patient (e.g., *adjacent* and *distant* explants) used linear mixed-effects models, with patient included as a random effect to account for the resulting lack of independence between observations. Models based exclusively on a single, independent observation per patient used a fixed-effects approach instead. For analyses involving two or more covariates, a backward, AIC-guided stepwise selection procedure was applied: all biologically relevant covariates were initially included and then removed one at a time if their exclusion improved or did not worsen model fit, favoring the most parsimonious model that best explained the variability in protein expression. Cases with missing values for a given covariate were excluded from the corresponding analysis (complete-case basis). Model assumptions were assessed through residual diagnostics in all cases. When a covariate or interaction term was statistically significant, Tukey’s post hoc tests were performed to identify specific groups with significantly different means. Correlation analyses were performed using Pearson’s correlation test. Effect sizes and 95% confidence intervals are reported alongside *p*-values throughout the Results. For all the analyses, a significance level of 0.05 was adopted (α = 0.05), and *p*-values below this threshold were considered statistically significant. All statistical analyses were conducted using R (version 4.3.2) [19] and RStudio (version 2023.09.1) [20]. All experiments were performed at least three times. The number of observations underlying each model is provided in Table S3 of the Supplementary Material.

## 3. Results

### 3.1. Expression of Lipolytic Proteins in Breast Adipose Tissue Explants from Healthy Donors and Breast Cancer Patients

Analysis of key lipolytic proteins provides valuable insights into the functional state of adipocytes within the tumor microenvironment and their potential role in supplying energy to tumor cells. To assess the expression of critical components of the lipolytic machinery, we evaluated the levels of perilipin 1, ATGL, and HSL in *normal*, *adjacent*, and *distant* explants by Western blot (WB) analysis. The characteristics of the study subjects (healthy donors and breast cancer patients) are summarized in Table 1.

**Figure 1:**
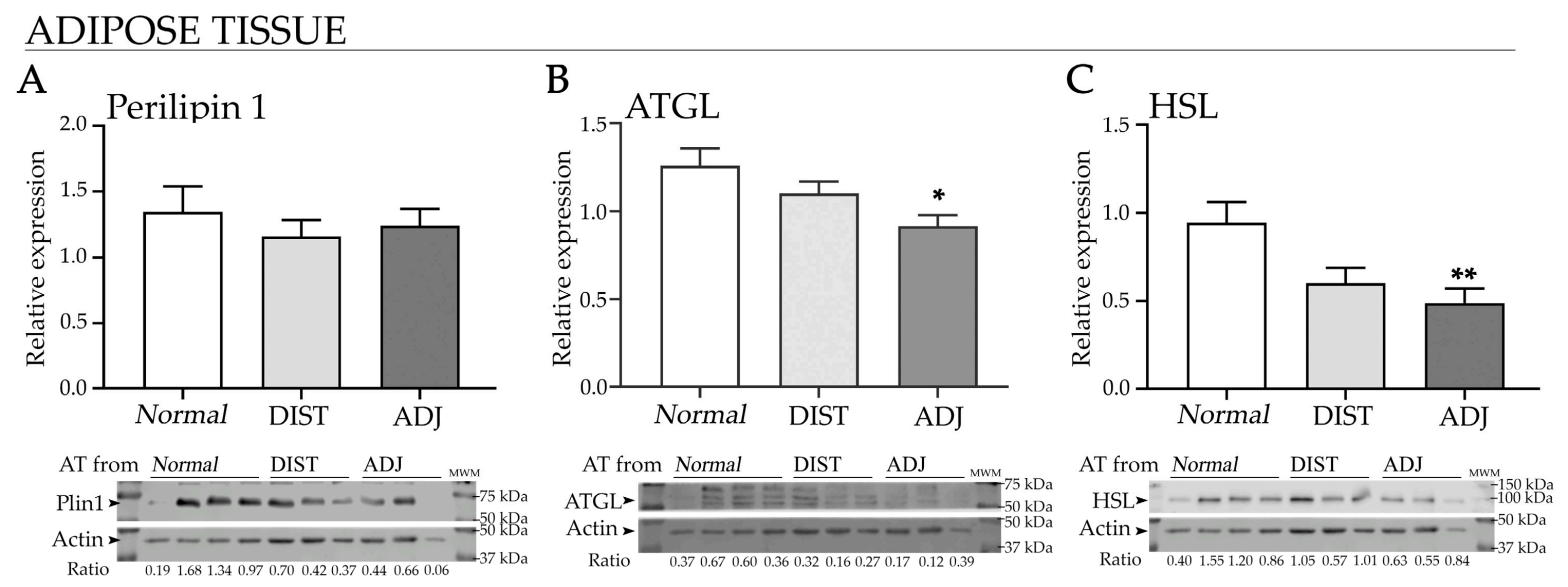
Expression of lipolytic proteins in human breast AT explants. Protein expression levels of perilipin 1 (**A**), ATGL (**B**), and HSL (**C**) were assessed by Western blot in *normal*, *adjacent*, and *distant* breast AT explants. Representative Western blots are shown. Densitometric analysis was performed on the corresponding bands indicated by arrows across all analyzed membranes. Ratios correspond to the normalized densitometric intensity of the representative bands shown. Data are presented as the mean ± SEM. * *p* < 0.05, ** *p* < 0.01. ADJ, *adjacent* breast explants (AT < 2 cm from the tumor); DIST, *distant* breast explants (AT > 2 cm from the tumor); MWM, molecular weight markers; Plin1, perilipin 1.

Perilipin 1 expression remained unchanged across all explants (Figure 1A). ATGL and HSL expression levels were lower in *adjacent* AT than in AT from *normal* controls (*p* < 0.05, 95% CI [0.059, 0.629] for ATGL, and *p* < 0.01, 95% CI [0.105, 0.807] for HSL) (Figure 1B,C). In addition, ATGL and HSL expression showed a trend toward lower levels in *distant* explants compared with *normal* explants (Figure 1B,C).

Since *normal*, *distant*, and *adjacent* explants originate from distinct biological contexts, their comparison provides a broad framework for evaluating the expression of lipolytic proteins across breast AT. Tumor proximity may influence the local AT microenvironment and the expression of proteins involved in lipid droplet regulation and lipolysis.

Although the healthy and breast cancer cohorts differed in some clinical characteristics—including age, menopausal status, and BMI, which may independently influence AT biology and the expression of lipolysis-associated proteins—the comparison provides an initial characterization of differences in perilipin 1, ATGL and HSL expression between AT from both cohorts. These differences should therefore be interpreted as descriptive associations rather than as evidence of tumor-specific effects.

To gain deeper insight into alterations in lipolytic protein expression within the TAAT microenvironment, subsequent analyses focused exclusively on the breast cancer patient cohort.

### 3.2. Influence of Breast Cancer Subtype and Clinical Characteristics on Lipolytic Protein Expression in the Adipose Tissue of the Tumor Microenvironment

Breast cancer heterogeneity presents a major challenge for accurate diagnosis, prognosis, and treatment [2,21]. Intrinsic tumor properties, as well as clinical factors, are associated with disease progression and severity [22,23]. The AT within the breast tumor microenvironment undergoes remodeling, characterized by changes in its cellular and molecular features. In this context, characterizing protein expression patterns in TAAT may provide insights into molecular alterations associated with tumor heterogeneity and clinical characteristics. Therefore, by analyzing the breast cancer cohort, we aimed to determine whether tumoral and clinical characteristics were associated with differences in the expression of key lipolytic proteins in AT within the breast tumor microenvironment.

We compared the expression of perilipin 1, ATGL, and HSL in breast AT explants using linear regression models that included clinical and pathological parameters (categorical and numerical covariates) (Table 2) in order to assess associations between tumor-related and patient-related characteristics and lipolytic protein expression within TAAT.

First, we analyzed the expression of lipolytic proteins in TAAT, which we defined as all explants from the tumor microenvironment (*adjacent* and *distant*), and subsequently in *adjacent* explants only.

**Figure 2:**
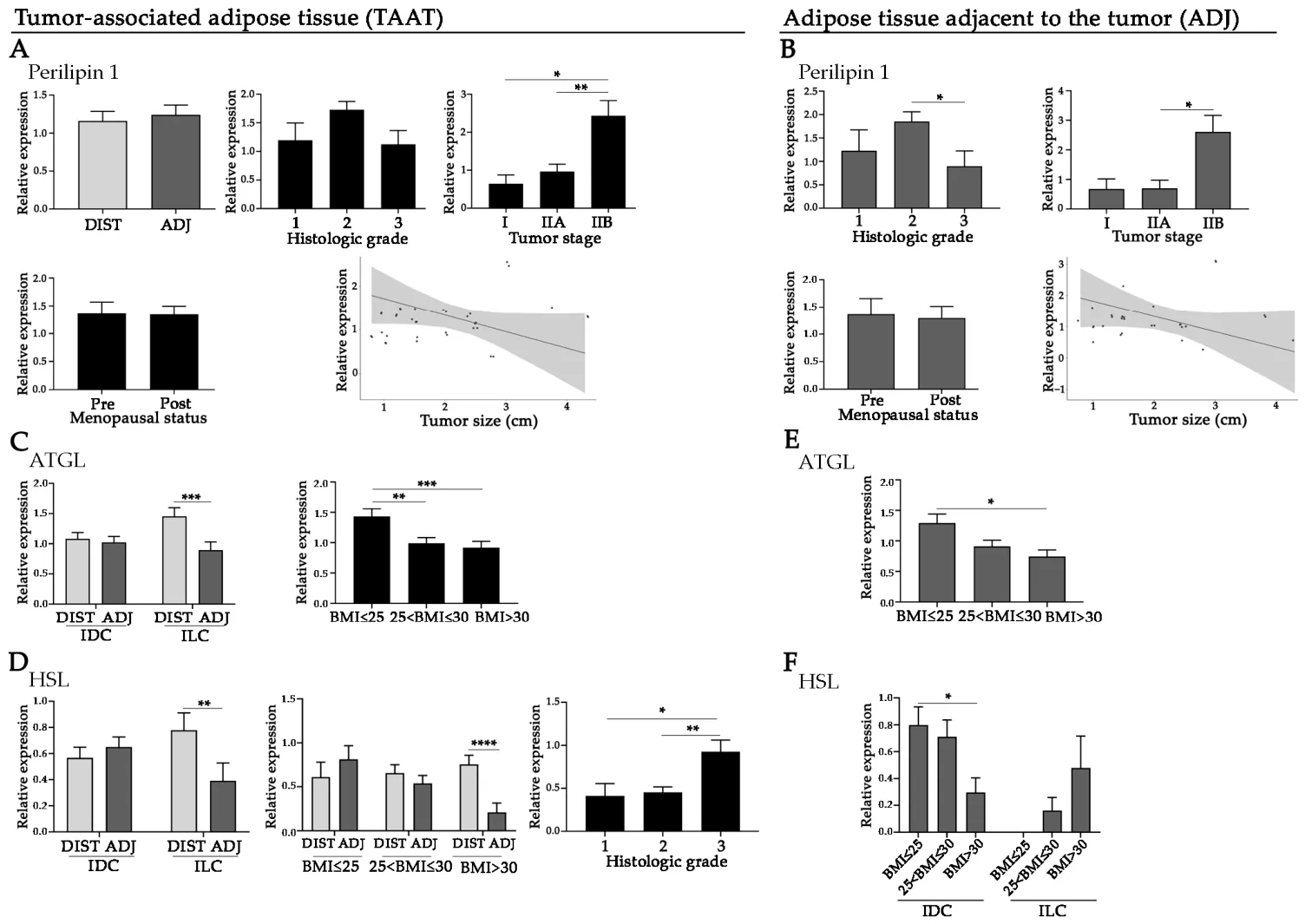
Lipolytic protein expression in AT explants from breast cancer patients. Estimated means of perilipin 1, ATGL and HSL expression by WB assays for covariates within the optimal linear regression model in TAAT ((**A**), (**C**) and (**D**), respectively) and *adjacent* explants ((**B**), (**E**) and (**F**), respectively). Actin was used as a loading control. Data are presented as the mean ± SEM. * *p* < 0.05, ** *p* < 0.01, *** *p* < 0.001, **** *p* < 0.0001. ADJ, *adjacent* breast explants (AT < 2 cm from the tumor); BMI, body mass index; DIST, *distant* breast explants (AT > 2 cm from the tumor); IDC, invasive ductal carcinoma; ILC, invasive lobular carcinoma; TAAT, tumor-associated adipose tissue.

The best-fitting linear regression model explaining the variation in perilipin 1 expression included proximity to the tumor, histologic grade, tumor stage, menopausal status, and tumor size. Perilipin 1 expression showed a trend toward higher levels in TAAT explants from patients with histologic grade 2 tumors compared with those from patients with grade 3 tumors (Figure 2A). Additionally, perilipin 1 expression was significantly higher in TAAT explants from patients with stage IIB breast cancer than those from patients with stage I (*p* < 0.05, 95% CI [−3.262, −0.317]) and IIA tumors (*p* < 0.01, 95% CI [−2.571, −0.372]) (Figure 2A). No significant differences were observed for the other covariates analyzed.

Next, we analyzed the influence of covariates on perilipin 1 expression in *adjacent* explants. The linear regression model included histologic grade, tumor stage, menopausal status, and tumor size. Perilipin 1 expression was significantly higher in *adjacent* explants from patients with histologic grade 2 tumors than in those from patients with grade 3 tumors (*p* < 0.05, 95% CI [0.053, 1.856]) (Figure 2B). Additionally, perilipin 1 levels were significantly higher in patients with stage IIB breast cancer than in those with stage IIA disease (*p* < 0.05, 95% CI [−3.480, −0.340]) and showed a trend toward lower levels in patients with stage I disease (*p* = 0.08, 95% CI [−4.052, 0.184]) (Figure 2B). No significant differences were observed for the other covariates included in the model.

The linear regression models for ATGL and HSL both included the interaction between proximity to the tumor and cancer subtype. The individual models also incorporated additional covariates: BMI for the ATGL model, and histologic grade plus the interaction between proximity to the tumor and BMI for the HSL model. Both proteins showed lower expression in *adjacent* than in *distant* explants from patients with invasive lobular carcinoma (ILC) (*p* < 0.001, 95% CI [−0.981, −0.138] for ATGL and *p* < 0.01, 95% CI [−0.721, −0.055] for HSL). In addition, ATGL expression was significantly lower in overweight (BMI between 25 and 30) and obese (BMI over 30) individuals (*p* < 0.01, 95% CI [0.138, 0.746] and *p* < 0.001, 95% CI [0.222, 0.811], respectively) (Figure 2C) in TAAT explants. HSL expression also decreased in *adjacent* explants from obese individuals (*p* < 0.05, 95% CI [0.121, 1.084]) and increased in patients with histologic grade 3 tumors compared to grade 1 (*p* < 0.05, 95% CI [−0.996, −0.029]) and grade 2 (*p* < 0.01, 95% CI [−0.825, −0.119]) (Figure 2D).

The linear regression models for *adjacent* explants included BMI for ATGL and the interaction between tumor subtype and BMI for HSL. ATGL expression was significantly lower in *adjacent* explants from obese patients than in those from normal-weight patients (*p* < 0.05, 95% CI [0.121, 0.991]) and showed a trend toward lower levels in overweight patients (*p* = 0.08, 95% CI [−0.041, 0.810]) (Figure 2E). HSL expression in *adjacent* explants was lower in obese patients with invasive ductal carcinoma (IDC) than in normal-weight (*p* < 0.05, 95% CI [0.058, 0.947]) and overweight individuals (*p* = 0.056, 95% CI [−0.007, 0.838]) (Figure 2F).

Despite the observed statistical differences, these results should be considered cautiously given the limited representation of specific groups, which precludes drawing definitive conclusions.

### 3.3. Heterogeneous Lipolytic Protein Expression in Adipose Tissue of the IDC Tumor Microenvironment

**Figure 3:**
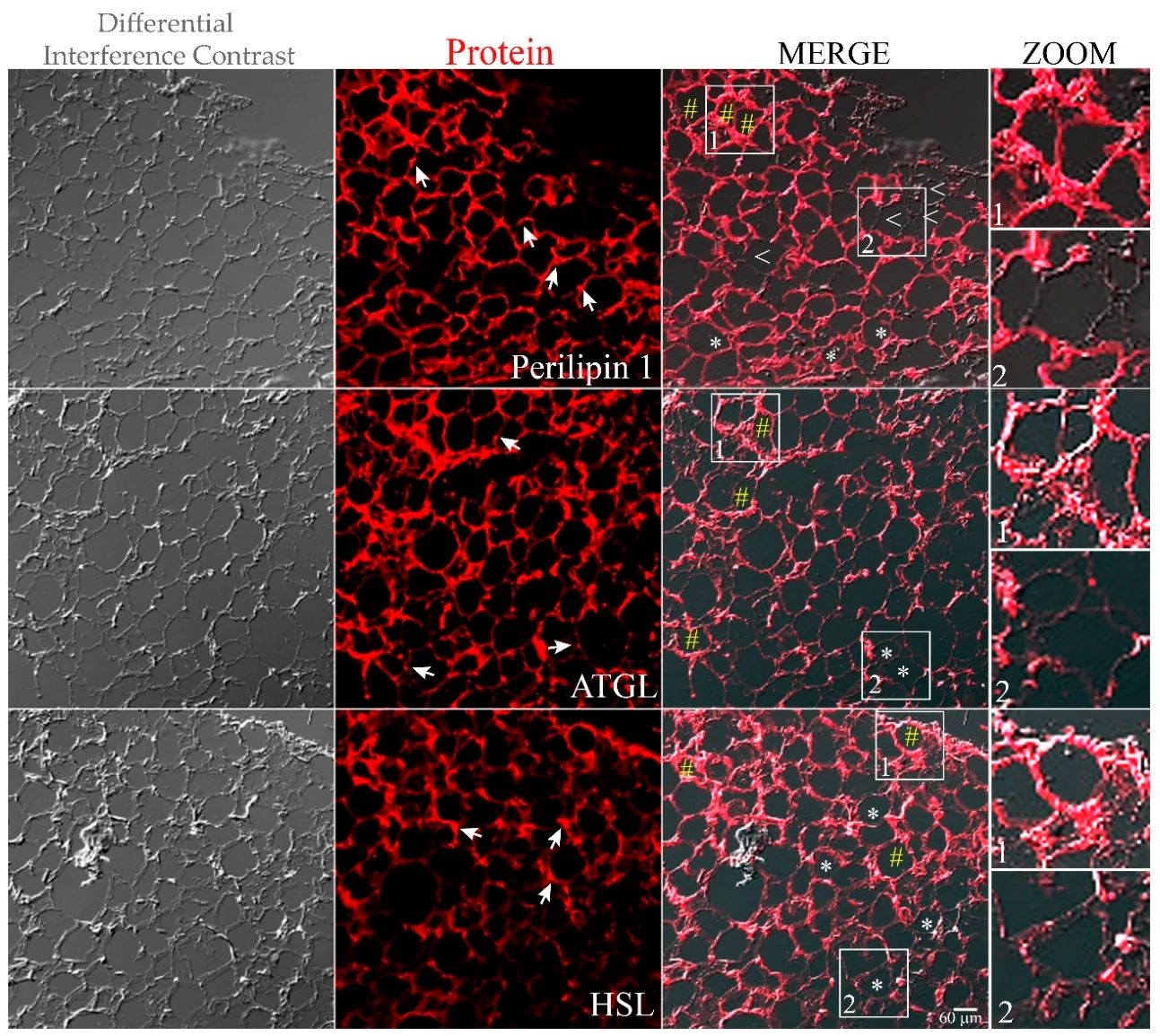
Heterogeneity of lipolytic protein expression in breast adipose explants. Representative images depicting the expression of perilipin 1 (**top panels**), ATGL (**middle panels**), and HSL (**bottom panels**) in breast AT of patients with IDC by IIF. White arrows indicate domains with a high concentration of the lipolytic proteins, arrowheads indicate adipocytes without a detected signal, asterisks highlight adipocytes with low fluorescence intensity and yellow number signs indicate adipocytes with high protein expression. Original magnification: ×100; scale bar: 60 µm; MERGE panels: Boxed areas 1 (indicating high fluorescence intensity) and 2 (indicating low fluorescence intensity or no detected signal) are magnified in the corresponding right panels (ZOOM). IDC, invasive ductal carcinoma.

We employed indirect immunofluorescence (IIF) to analyze the influence of covariates on the expression of lipolytic proteins in adipocytes within the tumor microenvironment of IDC, the most common type of breast cancer [24,25]. This approach enabled the subcellular localization and quantification of fluorescence intensity, providing insights into the distribution of proteins that are crucial for lipolysis [9,13] and their potential dependence on the analyzed covariates. Perilipin 1, ATGL, and HSL exhibited similar distribution patterns (Figure 3, protein panels).

This analysis revealed the heterogeneity of lipolytic protein expression within the tumor microenvironment by highlighting adipocytes with varying levels of perilipin 1, ATGL, and HSL expression (Figure 3, MERGE and ZOOM panels). These proteins were predominantly localized around lipid droplets, within regions exhibiting strong fluorescence signals. Explants from patients with IDC exhibited areas with varying fluorescence intensities, ranging from undetectable or low signal to strong signal for all proteins. This heterogeneous expression suggests that adipocytes exhibit distinct patterns of protein expression within the tumor microenvironment. The presence of distinct adipocyte clusters exhibiting high, intermediate, or undetectable protein expression may reflect spatial heterogeneity of adipocyte responses to tumor-derived signals. This variability further supports the concept that tumor–adipocyte interactions are associated with localized alterations in the expression of proteins involved in lipid droplet regulation and lipolysis.

### 3.4. Influence of IDC and Patient Characteristics on the Heterogeneous Expression of Lipolytic Proteins in the Adipose Tissue of the Tumor Microenvironment

We generated linear regression models that included categorical and numerical covariates (Table 3) to assess the impact of tumor heterogeneity and clinical characteristics on the fluorescence intensity of lipolytic proteins in the AT of the tumor microenvironment.

**Figure 4:**
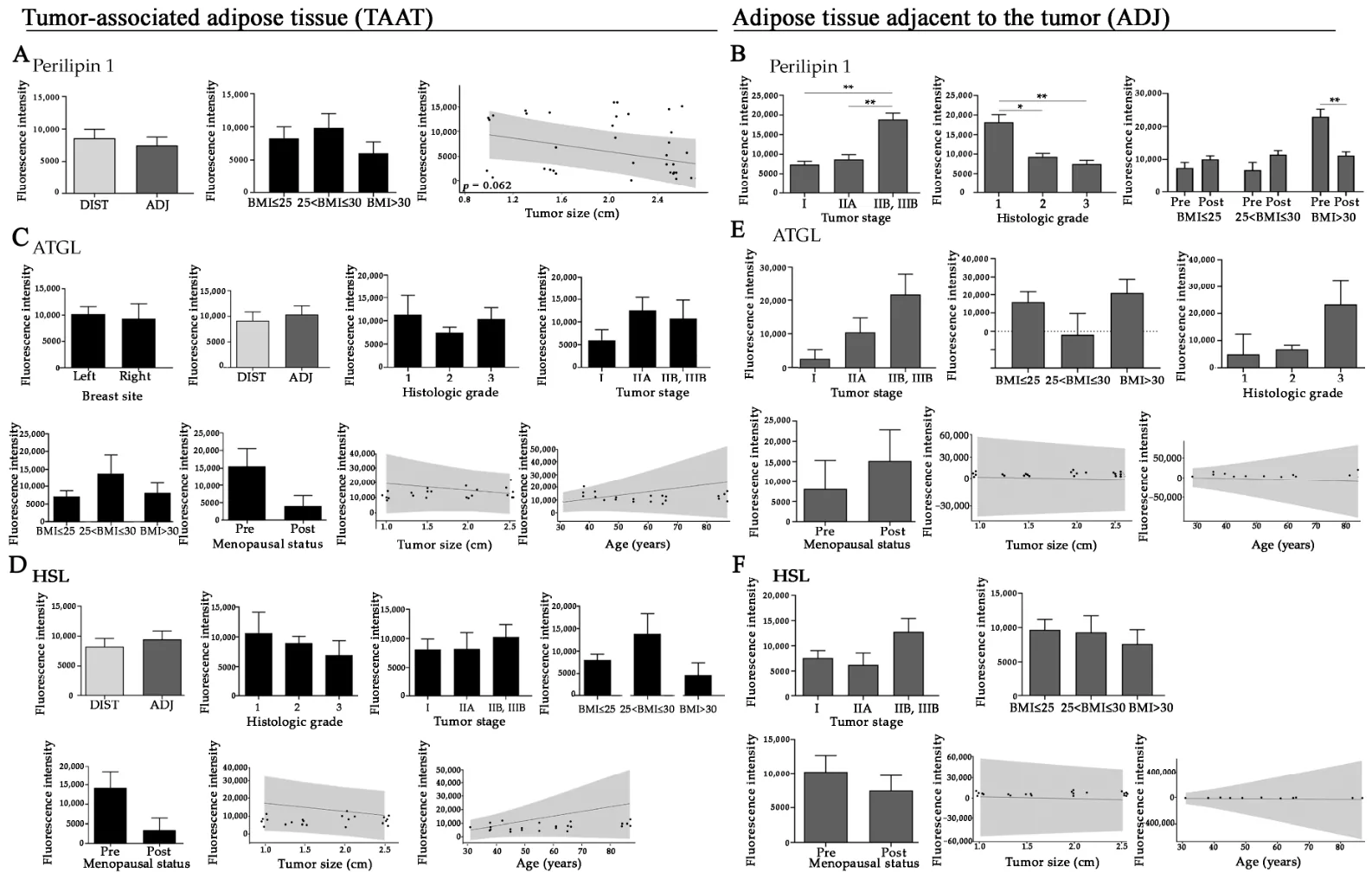
Lipolytic protein expression in breast adipocytes from patients with IDC. Estimated means of perilipin 1 (**A**,**B**), ATGL (**C**,**E**) and HSL (**D**,**F**) derived from IIF assays for covariates within the optimal linear regression model, categorized by TAAT ((**A**), (**C**) and (**D**), respectively) and *adjacent* explants ((**B**), (**E**) and (**F**), respectively). Fluorescence intensity was quantified in at least 50 adipocytes per explant using a custom-written ImageJ script. Data are presented as the mean ± SEM. * *p* < 0.05, ** *p* < 0.01. ADJ, *adjacent* breast explants (AT < 2 cm from the tumor); BMI, body mass index; DIST, *distant* breast explants (AT > 2 cm from the tumor); IIF, immunofluorescence; TAAT, tumor-associated adipose tissue.

The linear regression model for perilipin 1 fluorescence intensity in TAAT included the covariates tumor proximity, BMI, and tumor size, whereas the model for *adjacent* explants from patients with IDC included tumor stage, histologic grade, and the interaction between BMI and menopausal status. In TAAT explants, perilipin 1 fluorescence intensity was inversely associated with tumor size and showed no significant associations with the other covariates analyzed (Figure 4A). However, in *adjacent* explants, perilipin 1 fluorescence intensity was significantly higher in patients with stage IIB and IIIB tumors than in those with stage I (*p* < 0.01, 95% CI [−17,587, −5385]) and IIA tumors (*p* < 0.01, 95% CI [−15,465, −4899]) (Figure 4B). Additionally, perilipin 1 fluorescence intensity was higher in *adjacent* explants from patients with histologic grade 1 tumors than in those with grade 2 (*p* < 0.05, 95% CI [1787, 15,888]) and 3 tumors (*p* < 0.01, 95% CI [4779, 16,547]) and in obese (BMI > 30) premenopausal patients compared with obese postmenopausal patients (*p* < 0.01, 95% CI [2540, 20,901]) (Figure 4B). Despite the observed statistical differences, these results should be considered preliminary because of the limited representation of specific groups, particularly patients with histologic grade 1 tumors (*n* = 2), which limits the strength of any definitive conclusions.

The linear regression model for ATGL fluorescence intensity in TAAT from patients with IDC included the covariates breast site, tumor proximity, histologic grade, tumor stage, BMI, menopausal status, tumor size, and age. The regression model for HSL included the same covariates, except for breast site. Although ATGL fluorescence intensity did not differ significantly across the analyzed covariates, it showed a trend toward higher levels in premenopausal than in postmenopausal women with IDC (Figure 4C). HSL fluorescence intensity was also higher in the same group of patients (Figure 4D).

The linear regression models for lipase fluorescence intensity in adjacent explants from patients with IDC included histologic grade, tumor stage, BMI, menopausal status, tumor size, and age for ATGL, and the same covariates except for histologic grade for HSL. ATGL fluorescence intensity showed a trend toward higher levels in adipocytes from adjacent explants of grade 3 tumors and stage IIB or IIIB tumors (Figure 4E). HSL fluorescence intensity showed a similar trend when tumor stage was analyzed in adjacent explants from patients with IDC (Figure 4F).

### 3.5. Correlation of Lipolytic Protein Expression in Adipose Tissue Within the Tumor Microenvironment

**Figure 5:**
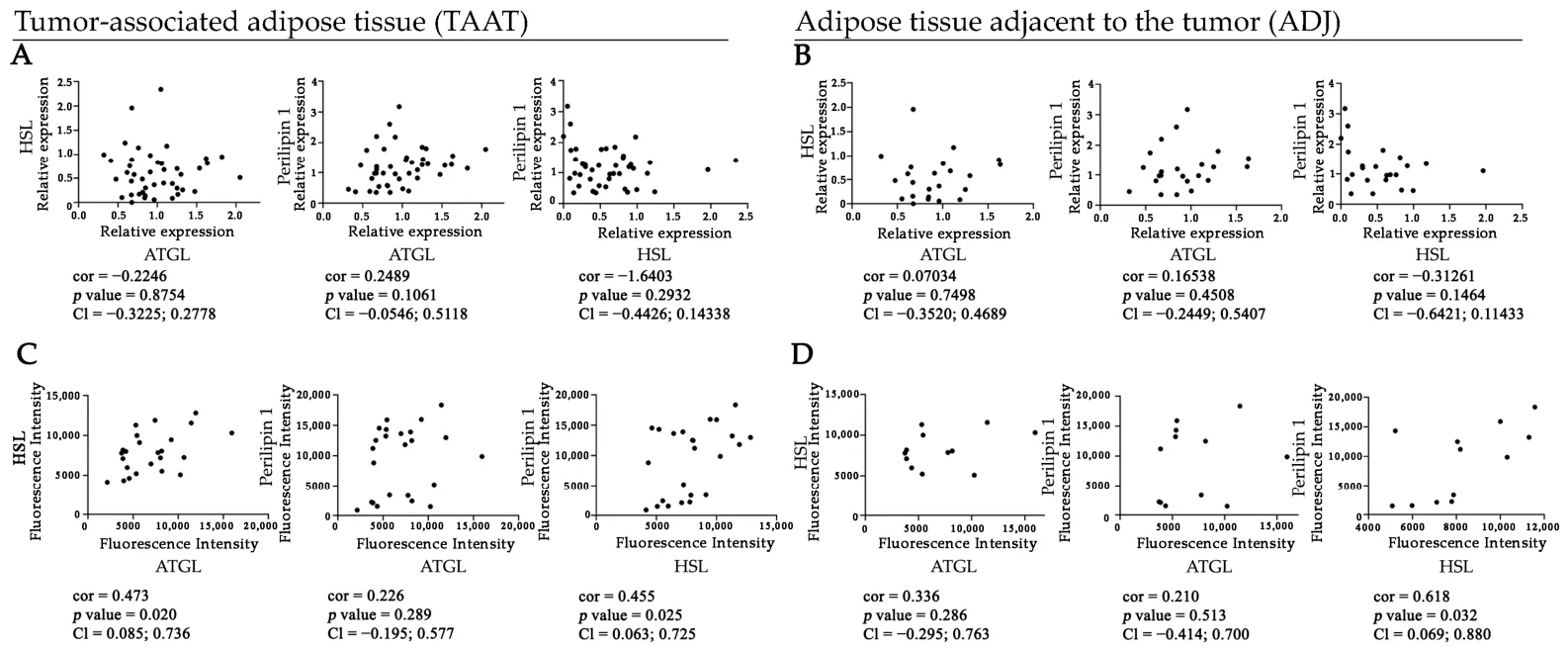
Correlation of lipolytic protein expression in the breast tumor adipose microenvironment. Scatter plots illustrate pairwise associations between perilipin 1, ATGL, and HSL expression levels. Relative protein expression determined by WB is shown for TAAT (**A**) and breast AT explants adjacent to the tumor (**B**). Fluorescence intensity per adipocyte determined by IIF is shown for TAAT (**C**) and breast AT explants adjacent to the tumor (**D**). cor, Pearson correlation coefficient; CI, confidence interval; IIF, immunofluorescence; TAAT, tumor-associated adipose tissue.

We performed correlation analyses based on protein expression in TAAT and *adjacent* explants to explore potential associations among key lipolytic proteins within the tumor microenvironment. While WB analysis did not reveal statistically significant correlations among perilipin 1, ATGL, and HSL regardless of the covariates considered (Figure 5A,B), IIF analysis provided additional insights. Specifically, we observed a significant positive correlation (*p* < 0.05) between ATGL and HSL expression in TAAT (Figure 5C). Perilipin 1 expression was also positively correlated with HSL expression in all explants, regardless of tumor proximity (Figure 5C,D).

These findings suggest that specific lipolytic proteins may exhibit coordinated expression patterns within AT in the breast cancer microenvironment, pointing to potential functional interactions among these proteins.

### 3.6. Exploring Sex-Dependent Variability in Lipolytic Protein Expression: A Preliminary Analysis in Male Breast Cancer

Male breast cancer is rare, accounting for approximately 1% of all breast cancer cases worldwide and 1% of all cancers affecting men [26]. It exhibits distinct metabolic, genetic, and epigenetic characteristics compared with female breast cancer [24,25,27]. Therefore, understanding sex-related metabolic differences and exploring whether lipolytic protein expression patterns differ according to sex could provide additional insights into the regulatory mechanisms governing lipolysis within the tumor microenvironment.

To explore potential sex-related differences in lipolytic protein expression, we assessed perilipin 1, ATGL, and HSL levels in TAAT and *adjacent* explants from male and female breast cancer patients with IDC, histologic grade 2, and stage IIA tumors. Although the number of male samples was scarce due to the rarity of male breast cancer, we considered this analysis relevant given the limited information available on sex-related variation in the expression of lipolysis-associated proteins in the breast tumor microenvironment. Perilipin 1 expression was comparable between male and female patients in both TAAT and *adjacent* explants (Figure S2). Lower ATGL and HSL expression was observed in male compared with female patients in both TAAT and *adjacent* explants (Figure S2).

Given the restricted sample size, these observations should be considered preliminary and interpreted cautiously. Larger cohorts will be required to determine whether the observed differences represent reproducible sex-related variation in the expression of lipolysis-associated proteins in breast TAAT.

## 4. Discussion

Lipid droplets serve as the primary energy storage site in eukaryotic cells and play a central role in lipid homeostasis. They are coated with perilipins, particularly perilipin 1, which regulate lipid metabolism [8,11]. Our study revealed that perilipin 1 expression remained unchanged when *normal* and tumor-associated explants were compared. However, when the analysis was restricted to the tumor context, perilipin 1 expression varied according to clinical patient characteristics and tumor-associated factors. Our findings showed increased perilipin 1 expression by WB in patients with stage IIB breast cancer, particularly in *adjacent* AT. Although cell types other than adipocytes within the tumor microenvironment may have contributed to this increase, IIF analysis confirmed higher perilipin 1 expression specifically in adipocytes from patients with advanced-stage IDC. These findings support a possible association between elevated perilipin 1 expression and poor breast cancer prognosis. Although perilipin 1 expression was also increased in patients with grade 1 tumors, this finding should be interpreted with caution due to the limited sample size. Nevertheless, the association between elevated perilipin 1 expression and poor prognosis is maintained, since tumor stage, rather than histologic grade, is a more reliable indicator of disease aggressiveness [28,29].

The role of peritumoral AT in the breast tumor microenvironment is not yet fully understood, and research in this area is challenging because of its complexity and heterogeneity. For example, premenopausal and postmenopausal women have distinct metabolic and hormonal profiles, particularly with respect to 17β-estradiol levels, which regulate perilipin 1 through transcriptional and post-translational mechanisms [30,31]. Moreover, the varied expression of perilipin 1 across different AT depots implies that its relationship to obesity remains controversial [32,33,34].

Our results indicate significantly increased perilipin 1 expression in *adjacent* AT from obese premenopausal women compared with obese postmenopausal women and other patient cohorts. This finding suggests a synergistic effect of premenopausal hormonal status and obesity on perilipin 1 upregulation within the tumor microenvironment. Given that both factors (obesity and hormonal status—in addition to tumor stage, as shown above) are associated with poorer breast cancer outcomes, worse prognosis, and reduced response to treatment [35,36,37,38], perilipin 1 emerges as a potential marker of disease progression. This interpretation is reinforced by the findings of Bombarda-Rocha et al. [39], who emphasized the role of perilipin 1 in lipid dysregulation-related diseases and highlighted the challenges associated with its pharmacological targeting alongside current treatments.

The expression of lipolytic proteins varies across different physiological and pathological contexts. This variability is at least partially influenced by estrogen receptors and 17β-estradiol, which directly and indirectly stimulate ATGL and HSL expressions in various AT depots and other tissues [30,40]. Expression of HSL is reduced in adipose tissue depots from obese individuals, leading to decreased catecholamine-induced lipolysis [41,42]. The role of ATGL in obesity remains disputed across different AT depots, suggesting complex mechanisms underlying the regulation of its gene expression [43,44,45].

Taken together, these findings hint that the decrease in lipolytic protein expression observed in our study may not be solely attributable to tumor-related effects but may also be influenced by differences in hormonal status and BMI. Consistent with this interpretation, premenopausal women showed a trend toward higher ATGL and HSL expression than postmenopausal women in our analysis of lipase expression within the breast tumor microenvironment of IDC. Furthermore, ATGL expression was lower in overweight and obese patients in TAAT, independent of tumor proximity, when BMI was considered as the only covariate. Expression of HSL was also significantly lower in obese patients, but only in *adjacent* explants, suggesting an effect of tumor proximity. The importance of considering tumor-driven effects and intrinsic patient characteristics when evaluating proteins involved in lipid homeostasis in TAAT is highlighted by the complex regulation of lipolytic protein expression within the tumor microenvironment, where systemic factors (e.g., hormonal status and BMI) and local tumor-induced alterations may both contribute to changes in protein expression. Supporting this interpretation, we observed lower ATGL and HSL expression in *adjacent* explants from patients with ILC than in *distant* explants. Patients with ILC have worse overall survival than those with IDC [46]. These findings further bolster the concept that tumor characteristics influence the tumor microenvironment, modulating lipolytic protein expression and potentially contributing to disease progression.

We examined potential correlations among key lipolytic proteins to explore the dysregulation of their expression within the breast tumor adipose microenvironment. Western blot analysis revealed no significant correlations among perilipin 1, ATGL, and HSL expression in AT. Nevertheless, a different pattern emerged when we focused exclusively on adipocytes within the tumor adipose microenvironment. Despite the variability arising from the metabolic heterogeneity of adipocytes, our findings suggest a positive correlation between perilipin 1 and HSL expression in adipocytes from the tumor adipose microenvironment (*adjacent* and *distant* explants). These findings agree with previous studies showing that adipocytes derived from perilipin 1-deficient mice exhibit reduced HSL levels, which insinuates a coordinated regulation of these lipolytic proteins [47]. Additionally, perilipin 1 and HSL undergo post-translational regulation through key signaling pathways, including PKA [8], which may be influenced by tumor-derived factors.

Our analysis also revealed a positive correlation between ATGL and HSL expression in TAAT. This finding is not unexpected, as both proteins exhibited similar expression patterns throughout our experiments, possibly because ATGL and HSL are regulated by shared transcriptional regulators of lipolysis-related genes [8]. However, we found no significant correlation between these proteins specifically in adipocytes adjacent to the tumor. This lack of association may reflect the influence of tumor proximity and other covariates, as well as the limited sample size, on protein expression.

While survival and recurrence analyses will be necessary to determine the clinical relevance of these alterations, understanding these changes provides insight into tumor–adipocyte crosstalk and emphasizes the need for further identification of potential metabolic biomarkers and therapeutic targets in breast cancer. The complexity of metabolic regulation within the tumor microenvironment highlights the importance of recognizing patterns of protein expression and their associations to refine prognostic models. Our correlation analyses revealed patterns in the scatter plots suggestive of cluster formation (Figure 5C, correlation analysis between perilipin 1 and lipases). However, further analyses incorporating covariates from linear regression and principal component analysis (PCA) models did not identify any clear explanatory variables. Recent studies examining germline mutational profiles in breast cancer have demonstrated substantial genetic heterogeneity and explored associations between molecular alterations and clinicopathological characteristics, highlighting the importance of considering biological and clinical heterogeneity when interpreting associations in patient cohorts [48]. Thus, unmeasured molecular or patient-related factors may have also contributed to the variability observed in our study. Although these clusters may not be random, their biological relevance remains uncertain, underscoring the need for larger cohorts and additional covariates relevant to breast cancer biology.

In addition, given the multifactorial nature of the tumor–adipocyte crosstalk, the alterations in protein expression observed in our findings may also be influenced by mechanisms beyond those examined in the present study. In particular, inflammatory mediators, adipokines and their associated signaling pathways, and epigenetic regulation may contribute to the regulation of lipolysis-associated proteins within the tumor microenvironment. Integrating these complementary mechanisms with protein expression profiles and relevant patient and tumor characteristics may therefore provide a more comprehensive understanding of the tumor-supportive adipose microenvironment and help identify candidate biomarkers and potential therapeutic targets.

The regulatory network involving perilipin 1, ATGL, and HSL plays a central role in lipid metabolism and mobilization within adipocytes. Our findings showed a marked decrease in ATGL and HSL expression in *adjacent* compared with *normal* explants, suggesting downregulation of these lipases in the tumor context. Nonetheless, a limitation of this study is that the cohorts available for comparative analysis differed in age, BMI, and menopausal status: *normal* samples were obtained predominantly from younger, normal-weight, premenopausal individuals, whereas tumor AT samples were obtained from older, postmenopausal, overweight, and obese individuals. These factors are known to influence lipase expression in AT depots outside the tumor context. This indicates that the comparison between AT from breast cancer patients and healthy controls should be interpreted with caution, and that the observed differences in ATGL and HSL between normal and TAAT cannot be attributed exclusively to tumor-related effects. Although further investigation of these variables is warranted, the primary objective of our study was to evaluate alterations in lipolytic protein expression specifically within the tumor microenvironment.

Metabolic alterations within the tumor microenvironment may not be uniform across all patient populations. For instance, male breast cancer exhibits distinct biological characteristics that require added research. In our exploratory analysis, we observed lower ATGL, and HSL expression in AT explants from male patients, concordant with previous studies [34]. These differences were observed in both TAAT and *adjacent* explants, suggesting a sex-dependent influence of the tumor microenvironment on lipase expression. Due to the limited availability of male samples in our study, additional studies are needed to validate these findings and elucidate potential sex-specific metabolic adaptations within the breast cancer microenvironment.

Moreover, as stated in the Introduction, lipolysis is primarily regulated at the post-translational level through phosphorylation events. In this study, we assessed the total expression of lipolytic proteins but not their phosphorylation status. This approach provides insights into the regulation of lipolytic protein expression and the availability of key proteins involved in lipolysis; however, our findings cannot be interpreted as a direct measure of lipolytic activity. Future studies should address this limitation by analyzing the phosphorylation status of HSL and perilipin 1, as well as upstream signaling pathways, including PKA and ERK. Functional assays of glycerol and fatty acid release, as well as measurement of lipase activity, should be performed. Such analyses will allow us to draw functional conclusions regarding the regulation of lipolysis within the tumor microenvironment, beyond the metabolic alterations observed in this study, which are based solely on changes in protein expression.

We recognize some additional limitations in this study. First, the sample size was relatively small; consequently, we applied appropriate statistical models that account for unbalanced datasets and carefully verified that all underlying assumptions were met. Although a larger sample would improve the generalizability of our findings, the associations we observed remain statistically valid and biologically meaningful within the context of our dataset. Therefore, although this study does not support population-wide inferences and is exploratory in scope, our results provide informative estimates and valuable insights into the alterations in the expression of lipolysis-associated proteins in TAAT. Rather than confirming pre-established hypotheses, these findings should be considered hypothesis-generating and seen as a strong foundation for future research.

## 5. Conclusions

Our findings indicate that breast TAAT is characterized by alterations in the expression of key proteins involved in lipid droplet regulation and lipolysis. Importantly, higher perilipin 1 expression was associated with selected clinicopathological features related to breast cancer aggressiveness, including advanced tumor stage, obesity, and premenopausal status. In contrast, ATGL and HSL expression were reduced in specific patient subgroups and tumor contexts, particularly in *adjacent* AT, obese patients, and invasive lobular carcinoma. These findings insinuate that the expression of lipolysis-associated proteins in the breast TAAT is influenced by both tumor proximity and clinicopathological characteristics.

Nevertheless, given the relatively limited sample size, particularly within individual subgroups, associations observed within those groups should be considered exploratory. Accordingly, perilipin 1, ATGL, and HSL may represent candidate markers of alterations in TAAT, and their association with aggressive clinicopathological features warrants further investigation.

## Figures and Tables

**Table 1:** Summary of the study subjects. Descriptive statistics for the studied covariates, from patients with hormone receptor-positive breast carcinoma without preoperative treatment (n = 23), and healthy women (*normal*, n = 11), included in the study of the expression of lipolytic proteins in breast AT explants.

Parameter	Healthy Women	Breast Cancer Patients	Overall
Age, years			
Mean (SD)	33.1 (12.4)	61.3 (13.9)	52.2 (18.9)
Median [Min, Max]	34.0 [17.0, 60.0]	64.0 [31.0, 84.0]	55.5 [17.0, 84.0]
	N (%)	N (%)	N (%)
Sex (%)			
Female	11 (100)	19 (82.6)	30 (88.2)
Male	n.d.	4 (17.4)	4 (11.8)
Menopausal status			
Pre	10 (90.9)	5 (21.7)	15 (44.1)
Post	1 (9.1)	14 (60.9)	15 (44.1)
Missing	n.d.	4 (17.4)	4 (11.8)
BMI, kg/m^2^			
≤25	9 (81.9)	4 (17.4)	13 (38.2)
25 < BMI ≥ 30	2 (18.1)	10 (43.5)	12 (35.3)
>30	n.d.	9 (39.1)	9 (26.5)

BMI: Body mass index; n.d.: no data.

**Table 2:** Clinical and pathological characteristics of all patients included in the analysis of lipolytic protein expression in breast AT explants by Western blot (*n* = 23 patients).

Parameter	
Age at diagnosis, years	
Mean (SD)	61.3 (13.9)
Median [Min, Max]	64.0 [31.0, 84.0]
Tumor size, cm	
Mean (SD)	2.09 (0.929)
Median [Min, Max]	2.00 [0.800, 4.30]
	N (%)
Menopausal status at diagnosis	
Pre	5 (21.7)
Post	14 (60.9)
Missing	4 (17.4)
BMI, kg/m^2^	
≤25	4 (17.4)
25 < BMI ≥ 30	10 (43.5)
>30	9 (39.1)
Tumor type	
IDC	18 (78.3)
ILC	5 (21.7)
Tumor stage	
I	9 (49.1)
IIA	11 (47.8)
IIB	3 (13.0)
Histologic grade	
1	2 (8.7)
2	16 (69.6)
3	5 (21.7)
Breast site	
Left	8 (34.8)
Right	15 (65.2)

BMI, body mass index; IDC, invasive ductal carcinoma; ILC, invasive lobular carcinoma.

**Table 3:** Clinical and pathological characteristics from patients with IDC included in the study of the expression of lipolytic proteins in breast AT explants by IIF (*n* = 19 patients).

Parameter	
Age at diagnosis, years	
Mean (SD)	61.3 (17.5)
Median [Min, Max]	63.0 [31.0, 87.0]
Tumor size, cm	
Mean (SD)	1.86 (0.678)
Median [Min, Max]	2.00 [0.500, 2.70]
	N (%)
Sex	
Female	17 (89.5)
Male	2 (10.5)
Menopausal status at diagnosis	
Pre	6 (31.6)
Post	11 (57.9)
Missing	2 (10.5)
BMI, kg/m^2^	
≤25	7 (36.8)
25 < BMI ≥ 30	5 (26.3)
>30	7 (36.8)
Tumor stage	
I	7 (36.8)
IIA	9 (47.4)
IIB, IIIB	3 (15.8)
Histologic grade	
1	2 (10.5)
2	11 (57.9)
3	6 (31.6)
Breast site	
Left	9 (47.4)
Right	10 (52.6)

BMI, body mass index.

## Data Availability

The datasets underlying the results of this study are included in the article and its Supplementary Files and are available from the corresponding author upon reasonable request.

## Supplementary Materials

The following supporting information can be downloaded at https://www.mdpi.com/article/10.3390/cancers18183001/s1, Figure S1: Original Western blot images showing protein expression of lipolytic proteins (Plin1, HSL and ATGL) in human breast AT explants; Figure S2: Expression of lipolytic proteins in AT explants from breast cancer patients by sex; Table S1: Summary of healthy donors information; Table S2: Summary of breast cancer patients information; Table S3a: Number of patients per protein and tissue group—healthy women vs. breast cancer patients; Table S3b: Number of patients per protein—tumor characterization by Western blot; Table S3c: Number of patients per protein—tumor characterization by indirect immunofluorescence (IDC patients only).

## Abbreviations

The following abbreviations are used in this manuscript:

AT	adipose tissue
ATGL	adipose triglyceride lipase
BMI	body mass index
HSL	hormone-sensitive lipase
IDC	invasive ductal carcinoma
IIF	indirect immunofluorescence
ILC	invasive lobular carcinoma
PKA	protein kinase A
Plin1	perilipin 1
TAAT	tumor-associated adipose tissue
TBS	tris-buffered saline
WB	Western blot
