# Supplementary material for: Altered Lipolytic Protein Expression in Tumor-Associated Adipose Tissue Correlates with Aggressive Breast Cancer Features
Source: Cancers (Basel). 2026 Sep 16;18(18):3001. doi: 10.3390/cancers18183001 (PMC13605504; doi:10.3390/cancers18183001)
Supplement: Supplementary file 1 [file cancers-18-03001-s001.zip › Supplementary Figure S1.pdf]

Supplementary File S1 (Figure S1)

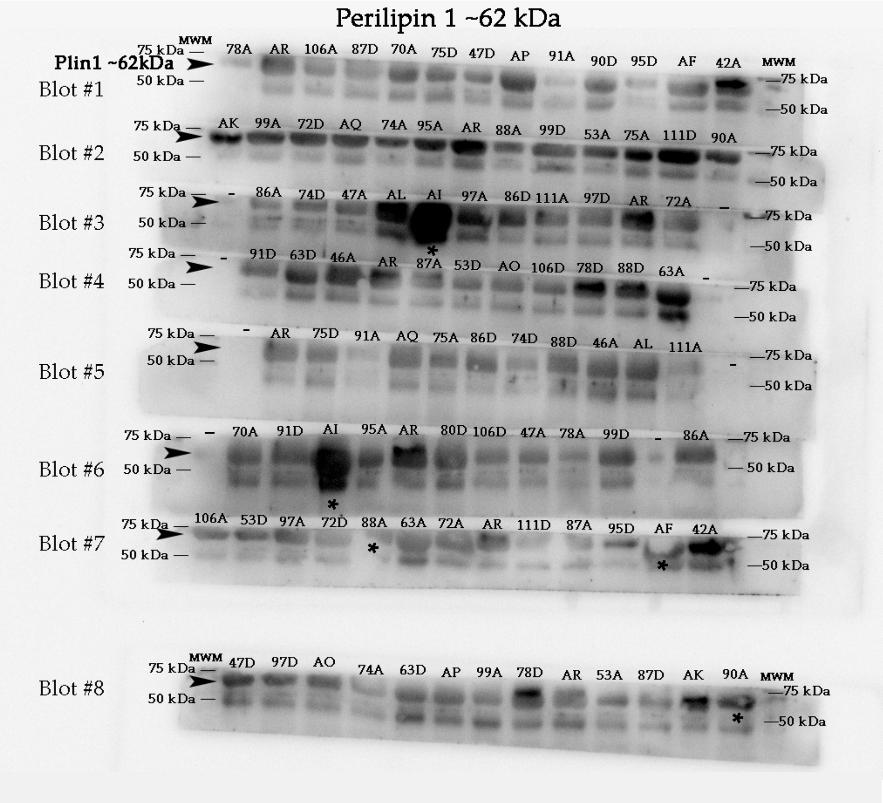

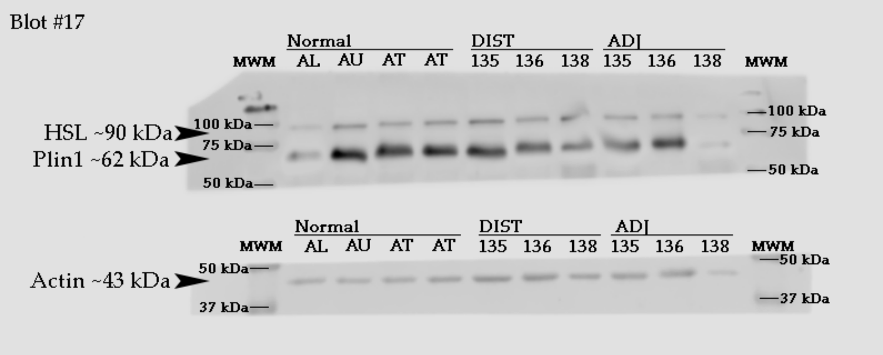

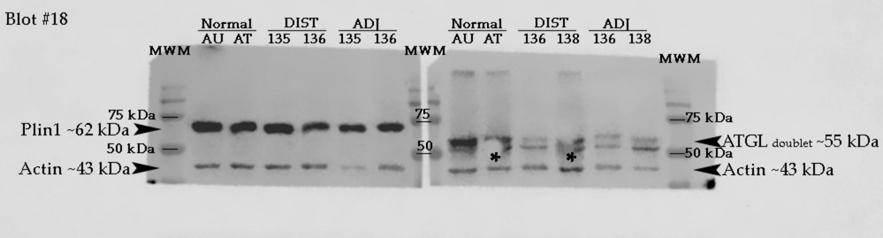

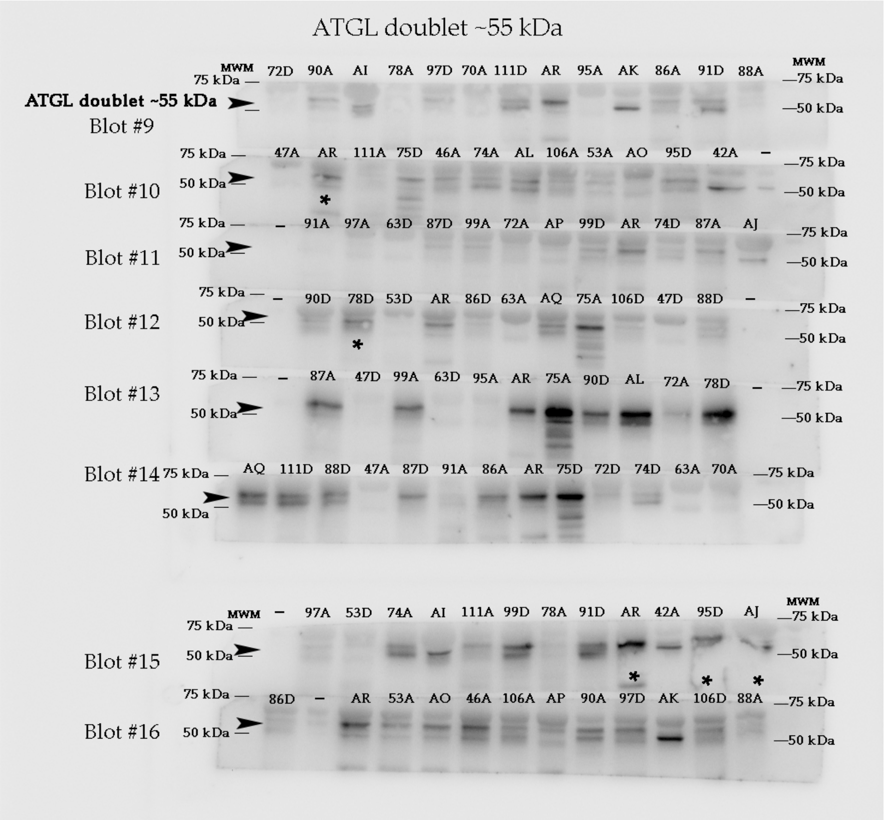
ATGL doublet ~55 kDa
MWM 72D 90A AI 78A 97D 70A 111D AR 95A AK 86A 91D 88A MWM
ATGL doublet ~55 kDa
Blot #9
47A AR 111A 75D 46A 74A AL 106A 53A AO 95D 42A –
Blot #10
– 91A 97A 63D 87D 99A 72A AP 99D AR 74D 87A AJ
Blot #11
– 90D 78D 53D AR 86D 63A AQ 75A 106D 47D 88D –
Blot #12
– 87A 47D 99A 63D 95A AR 75A 90D AL 72A 78D –
Blot #13
Blot #14
AQ 111D 88D 47A 87D 91A 86A AR 75D 72D 74D 63A 70A
MWM – 97A 53D 74A AI 111A 99D 78A 91D AR 42A 95D AJ MWM
Blot #15
86D – AR 53A AO 46A 106A AP 90A 97D AK 106D 88A
Blot #16
75 kDa
50 kDa

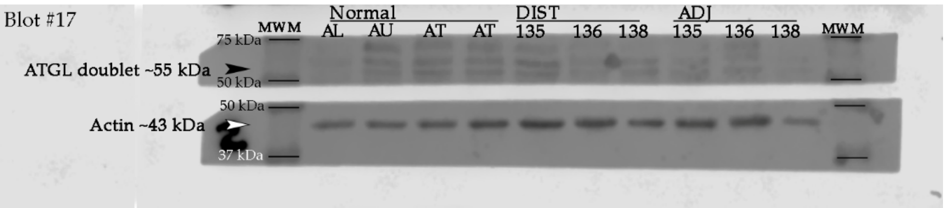
Blot #17
Normal
DIST
ADJ
MWM AL AU AT AT 135 136 138 135 136 138 MWM
75 kDa
ATGL doublet ~55 kDa
50 kDa
Actin ~43 kDa
37 kDa

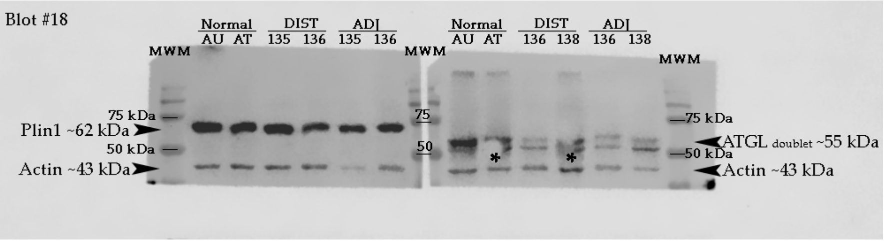
Blot #18
Normal DIST ADJ
AU AT 135 136 135 136
Normal DIST ADJ
AU AT 136 138 136 138
MWM
75 kDa
Plin1 ~62 kDa
50 kDa
Actin ~43 kDa
ATGL doublet ~55 kDa
Actin ~43 kDa

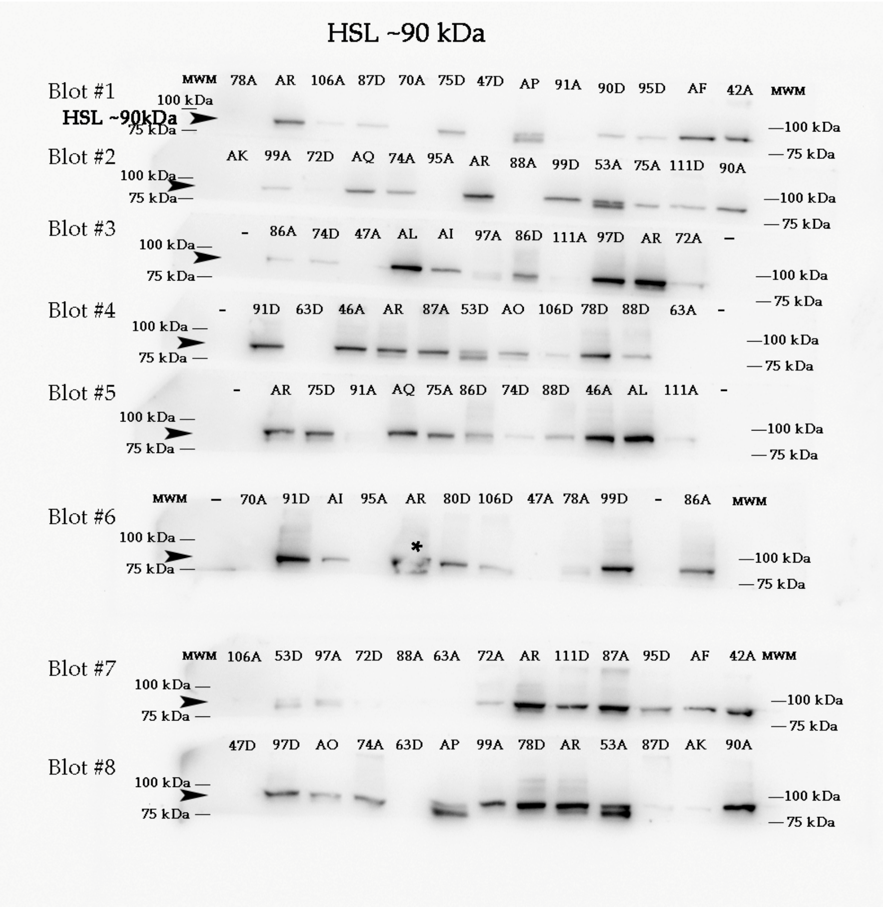
HSL ~90 kDa
Blot #1
MWM 78A AR 106A 87D 70A 75D 47D AP 91A 90D 95D AF 42A MWM
HSL ~90kDa
100 kDa
75 kDa
—100 kDa
—75 kDa
Blot #2
AK 99A 72D AQ 74A 95A AR 88A 99D 53A 75A 111D 90A
Blot #3
- 86A 74D 47A AL AI 97A 86D 111A 97D AR 72A -
Blot #4
- 91D 63D 46A AR 87A 53D AO 106D 78D 88D 63A -
Blot #5
- AR 75D 91A AQ 75A 86D 74D 88D 46A AL 111A -
Blot #6
MWM - 70A 91D AI 95A AR 80D 106D 47A 78A 99D - 86A MWM
*
Blot #7
MWM 106A 53D 97A 72D 88A 63A 72A AR 111D 87A 95D AF 42A MWM
Blot #8
47D 97D AO 74A 63D AP 99A 78D AR 53A 87D AK 90A

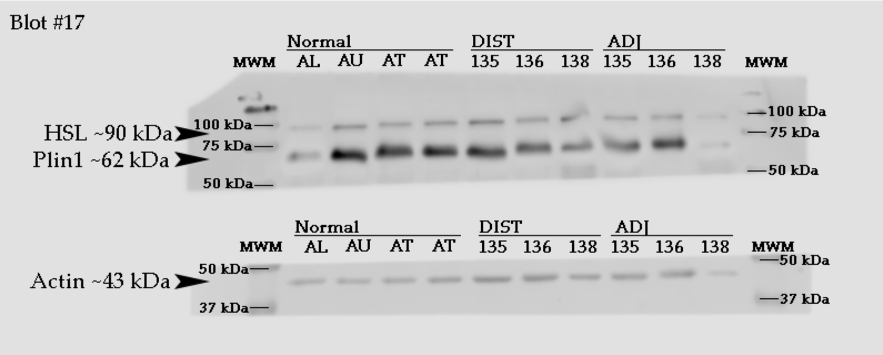
Blot #17
Normal
DIST
ADJ
MWM AL AU AT AT 135 136 138 135 136 138 MWM
HSL ~90 kDa
Plin1 ~62 kDa
100 kDa
75 kDa
50 kDa
Actin ~43 kDa
50 kDa
37 kDa

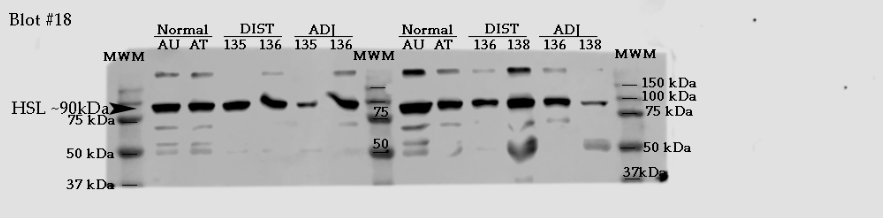
Blot #18
Normal
DIST
ADJ
AU AT 135 136 135 136
Normal
DIST
ADJ
AU AT 136 138 136 138
MWM
MWM
MWM
HSL ~90kDa
75 kDa
50 kDa
37 kDa
75
50
150 kDa
100 kDa
75 kDa
50 kDa
37kDa

Actin ~43 kDa

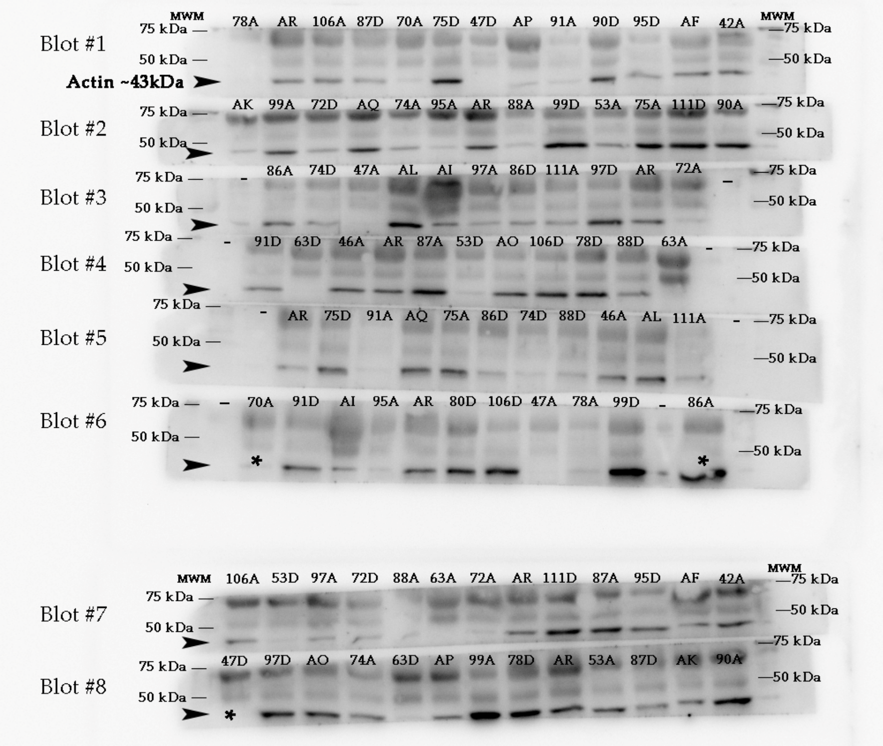

Actin ~43 kDa

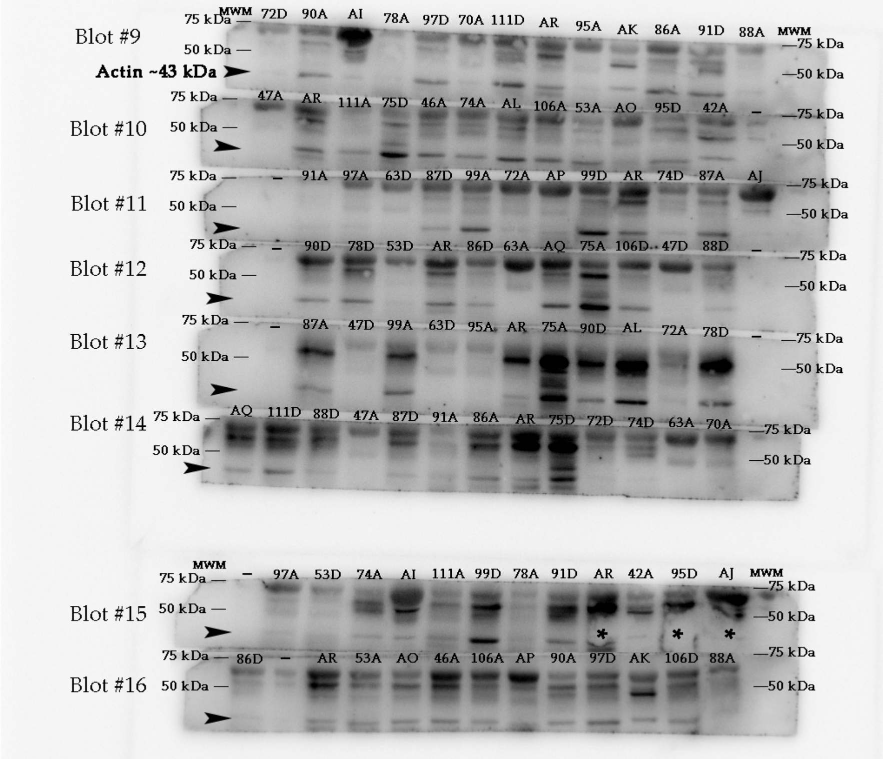

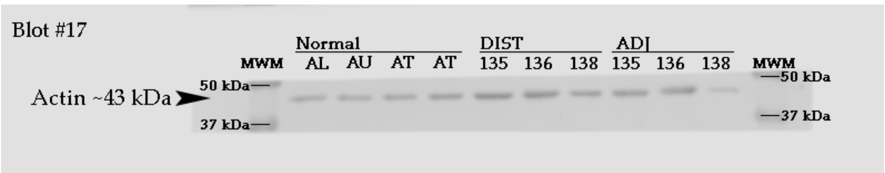

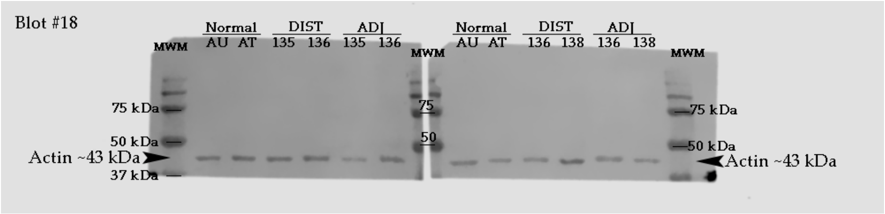

Supplementary Figure S1. Original Western blot images showing protein expression of lipolytic proteins (Perilipin 1, HSL and ATGL) in human breast adipose tissue explants. Actin served as loading controls. Adipose explants used to assay: n *adjacent* = 23, n *distant* = 20, n *normal* = 10. Numbers followed by A indicate *adjacent* breast adipose tissue explants, numbers followed by D indicate *distant* breast adipose tissue explants, and two-letter codes indicate breast adipose tissue explants from healthy women. For additional information on healthy donors and breast cancer patients, see Tables S1 and S2. Arrows indicate the specific protein bands that were quantified: Plin1 band (~62 kDa), ATGL doublet (~55 kDa), HSL band (~90 kDa), and actin band (~43 kDa). Perilipin 1 and HSL detection was performed on ten independent blots (blots 1-8, 17, and 18); ATGL detection, on ten independent blots (blots 9-18); actin detection, on the following blots: 1-18. According to the migration pattern of the molecular weight markers, membranes were sectioned horizontally, enabling the separate immunodetection of high- and medium-molecular-weight. ADJ, *adjacent* breast explants (AT < 2 cm from the tumor); DIST, *distant* breast explants (AT > 2 cm from the tumor); MWM, molecular weight markers; Plin1, perilipin 1; (*) Not considered.
