# Supplementary material for: Altered Lipolytic Protein Expression in Tumor-Associated Adipose Tissue Correlates with Aggressive Breast Cancer Features
Source: Cancers (Basel). 2026 Sep 16;18(18):3001. doi: 10.3390/cancers18183001 (PMC13605504; doi:10.3390/cancers18183001)
Supplement: Supplementary file 1 [file cancers-18-03001-s001.zip › Supplementary Figure S2.pdf]

Supplementary File S2 (Figure S2)

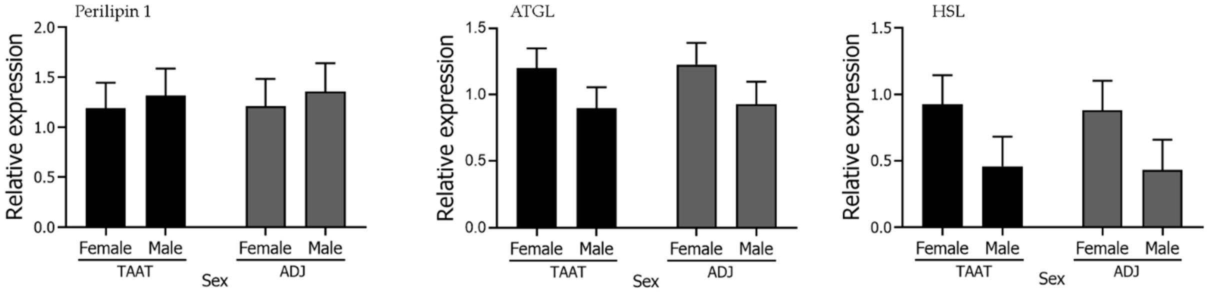

Figure S2 Expression of lipolytic proteins in AT explants from breast cancer patients by sex. Estimated means of Plin-1, ATGL and HSL expression by WB assays in TAAT and *adjacent* explants from patients with IDC, histologic grade 2, and stage IIA tumors. Actin was used as a loading control. Data are presented as the mean ± SEM (*nMale* = 4, and *nFemale* = 5). ADJ, adjacent breast explants (AT < 2 cm from the tumor); IDC, invasive ductal carcinoma; TAAT, tumor-associated adipose tissue.
