# Supplementary material for: Altered Lipolytic Protein Expression in Tumor-Associated Adipose Tissue Correlates with Aggressive Breast Cancer Features
Source: Cancers (Basel). 2026 Sep 16;18(18):3001. doi: 10.3390/cancers18183001 (PMC13605504; doi:10.3390/cancers18183001)
Supplement: Supplementary file 1 [file cancers-18-03001-s001.zip › Supplementary Table S1.pdf]

Table S1: Summary of healthy donors information

Healthy donors				
**ID code for AT Explants from donors (n = 11)**	**Age, years**	**BMI, kg/m^2**	**Sex**	**Menopausal status**
AF	34	25.4	Female	Pre
AI	36	17.6	Female	Pre
AJ	25	22.6	Female	Pre
AK	29	21.4	Female	Pre
AL	17	24.2	Female	Pre
AO	21	20.3	Female	Pre
AP	60	20.8	Female	Post
AQ	40	18.3	Female	Pre
AR	35	21.5	Female	Pre
AT	45	26.6	Female	Pre
AU	22	21.9	Female	Pre

AT: Adipose tissue; BMI: body mass index; ID: Identification
