# Supplementary material for: Altered Lipolytic Protein Expression in Tumor-Associated Adipose Tissue Correlates with Aggressive Breast Cancer Features
Source: Cancers (Basel). 2026 Sep 16;18(18):3001. doi: 10.3390/cancers18183001 (PMC13605504; doi:10.3390/cancers18183001)
Supplement: Supplementary file 1 [file cancers-18-03001-s001.zip › Supplementary Table S2.pdf]

Table S2: Summary of breast cancer patients information

Breast cancer patients										
**ID code for AT Explants from patients (n = 23)**	**AT Explants (n = 43)**	**Age, years**	**BMI, Kg/m²**	**Sex**	**Menopausal status**	**Tumor type**	**Tumor stage**	**Histology grade**	**Breast site**	**Tumor size, cm**
42	ADJ	38	21.5	Female	Pre	IDC	IIA	2	Left	1.5
46	ADJ	49	26.6	Female	Pre	IDC	IIB	3	Right	3.8
47	ADJ<br>DIST	56	42.9	Female	Post	IDC	I	2	Left	1.2
53	ADJ<br>DIST	69	30.5	Female	Post	IDC	IIA	2	Left	2.4
63	ADJ<br>DIST	67	31.0	Female	Post	IDC	IIB	2	Right	3.0
70	ADJ	79	35.0	Male	Missing	IDC	IIA	2	Left	2.7
72	ADJ<br>DIST	64	30.0	Male	Missing	IDC	IIA	2	Right	2.5
74	ADJ<br>DIST	31	20.8	Female	Pre	IDC	I	2	Right	1.0
75	ADJ<br>DIST	65	32.0	Male	Missing	IDC	IIA	2	Left	3.0
78	ADJ<br>DIST	66	39.4	Female	Post	ILC	I	2	Right	1.5
86	ADJ<br>DIST	55	32.4	Female	Post	IDC	IIB	3	Right	4.3
87	ADJ<br>DIST	84	23.5	Female	Post	IDC	IIA	2	Right	2.5
88	ADJ<br>DIST	42	26.6	Female	Pre	ILC	IIA	2	Right	2.0
90	ADJ<br>DIST	45	23.4	Female	Pre	IDC	IIA	2	Right	2.5
91	ADJ<br>DIST	61	28.0	Female	Post	IDC	I	3	Right	1.5
95	ADJ<br>DIST	79	25.2	Female	Post	ILC	I	2	Right	1.5
97	ADJ<br>DIST	52	28.5	Female	Post	ILC	IIA	2	Left	1.3
99	ADJ<br>DIST	66	30.5	Female	Post	IDC	I	1	Right	0.8
106	ADJ<br>DIST	73	28.8	Female	Post	IDC	I	2	Right	2.0
111	ADJ<br>DIST	58	29.8	Female	Post	ILC	I	1	Left	1.0
135	ADJ<br>DIST	79	25.9	Male	Missing	IDC	IIA	2	Left	2.2
136	ADJ<br>DIST	60	35.7	Female	Post	IDC	I	3	Right	1.0
138	ADJ<br>DIST	72	37.3	Female	Post	IDC	IIA	3	Right	2.8

ADJ, adjacent to the tumor; AT: Adipose tissue; BMI: Body Mass Index; DIST, > 2 cm from the tumor; ID: Identification; IDC, invasive ductal carcinoma; ILC, invasive lobular carcinoma.
