# Supplementary material for: Altered Lipolytic Protein Expression in Tumor-Associated Adipose Tissue Correlates with Aggressive Breast Cancer Features
Source: Cancers (Basel). 2026 Sep 16;18(18):3001. doi: 10.3390/cancers18183001 (PMC13605504; doi:10.3390/cancers18183001)
Supplement: Supplementary file 1 [file cancers-18-03001-s001.zip › Supplementary Table S3.pdf]

Table S3a: Number of patients per protein and tissue group — healthy women vs. breast cancer patients

Protein	Healthy women (Normal)	Breast Cancer Patients (Adjacent)	Breast Cancer Patients (Distant)
Perilipin 1	10	23	20
HSL	10	21	18
ATGL	10	23	20

Table S3b: Number of patients per protein — tumor characterization by western blot

Protein	N patients (TAAT)	N patients (ADJ)
Perilipin 1	19	19
HSL	21	21
ATGL	23	23

Table S3c: Number of patients per protein — tumor characterization by indirect immunofluorescence (IDC patients only)

Protein	N patients (TAAT)	N patients (ADJ)
Perilipin 1	19	16
HSL	13	12
ATGL	13	12
